# Intermittent Sinus Pause/Asystole in the Setting of Anticholinergic Overdose

**DOI:** 10.7759/cureus.77959

**Published:** 2025-01-25

**Authors:** Raymond Kwong, Courtney Collins

**Affiliations:** 1 Emergency Medicine, Western Michigan University Homer Stryker M.D. School of Medicine, Kalamazoo, USA

**Keywords:** anticholinergic toxicity, bentyl overdose, dicyclomine toxicity/overdose, intermittent asystole, intermittent sinus pause

## Abstract

Anticholinergic toxicity typically presents with symptoms of cutaneous vasodilation, delirium, mydriasis, urinary retention, hyperthermia, anhidrosis, and tachycardia. This case report presents a 68-year-old female patient who exhibited some of these signs and symptoms after ingesting an unknown quantity of dicyclomine. However, she displayed one notable exception to the classic toxidrome. On hospital day 2, the patient experienced multiple incidences of prolonged sinus pause, culminating in witnessed asystole lasting 5-10 seconds. The patient continued to have numerous episodes of sinus pause that lasted 5-10 seconds each over the next two days. Treatment involved placement of multiple temporary transvenous pacemakers until the episodes of sinus pause eventually self-resolved, facilitating discharge home on hospital day 6. The patient recovered without any known complications. After considering alternative diagnoses such as sick sinus syndrome, electrolyte derangements, and intracranial hypertension, multiple hospital medical services ultimately attributed the arrhythmia to anticholinergic toxicity. There are no other documented cases of intermittent sinus pause associated with anticholinergic overdose. While rare, clinicians should consider anticholinergic toxicity as a potential cause of intermittent sinus pause, especially in patients taking anticholinergic medications. Increased clinical vigilance could impact treatment decisions, including potentially avoiding unnecessary procedures such as permanent pacemaker placement, if symptoms resolve with cessation of the offending agent.

## Introduction

In an era marked by rising medication use, accidental toxicities are increasingly common in emergency department visits [1,2]. It is estimated that approximately 15-20% of poisoning admissions are due to anticholinergic overdose [3]. This is unsurprising given that there are over 600 prescription and over-the-counter medications with some anticholinergic properties, including antihistamines, antidepressants, antiemetics, antipsychotics, antispasmodics, skeletal muscle relaxers, and medication for urinary incontinence [4]. Some plants advertised as "natural" remedies, such as Jimson weed (*Datura stramonium*) and deadly nightshade (*Atropa belladonna*), can also have anticholinergic effects [5]. Anticholinergic medications inhibit the activity of acetylcholine, primarily at muscarinic receptors located in smooth muscle, glands (sweat and salivary), the ciliary body of the eye, and the central nervous system [6]. Anticholinergic toxicity is often remembered by the mnemonic "mad as a hatter, blind as a bat, red as a beet, hot as a hare, dry as a bone, and full as a flask," representing delirium, mydriasis, cutaneous vasodilation, hyperthermia, anhidrosis, and urinary retention [7]. Symptoms can also include hypoactive bowel sounds (due to decreased gastrointestinal (GI) motility), tachycardia, dry mucous membranes, dysarthria, restlessness, irritability, and hallucinations [7]. We present the case of a patient who exhibited some of these symptoms after ingesting dicyclomine. However, there was one notable exception to the typical presentation. Rather than exhibiting tachycardia, this patient experienced intermittent sinus pause. Determining the etiology of the patient's arrhythmia was difficult as history was limited due to the patient's mental status and considering there are no other documented cases of anticholinergic-induced sinus pause/asystole. After considering alternative diagnoses such as sick sinus syndrome, electrolyte derangements, and intracranial hypertension, the arrhythmia was ultimately attributed to anticholinergic toxicity. This case report presents the first documented association between anticholinergic toxicity and intermittent sinus pause/asystole.

## Case presentation

**Figure 1:**
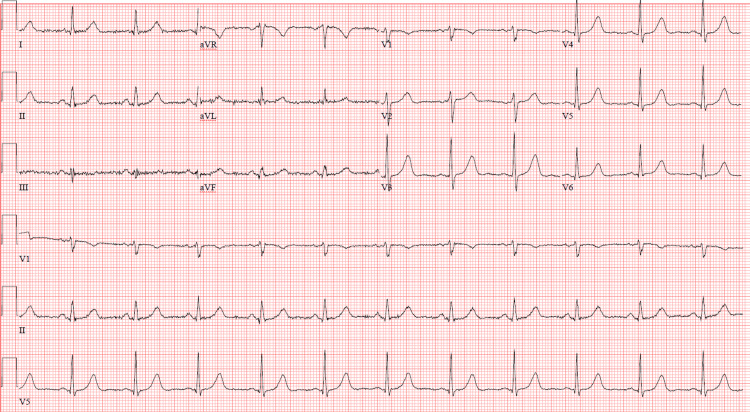
EKG post witnessed sinus pause and syncope EKG: electrocardiogram

**Figure 2:**
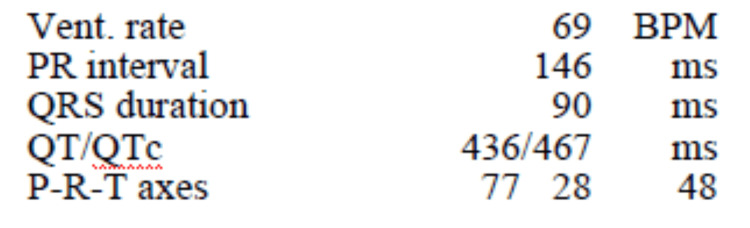
EKG intervals EKG: electrocardiogram

**Figure 3:**
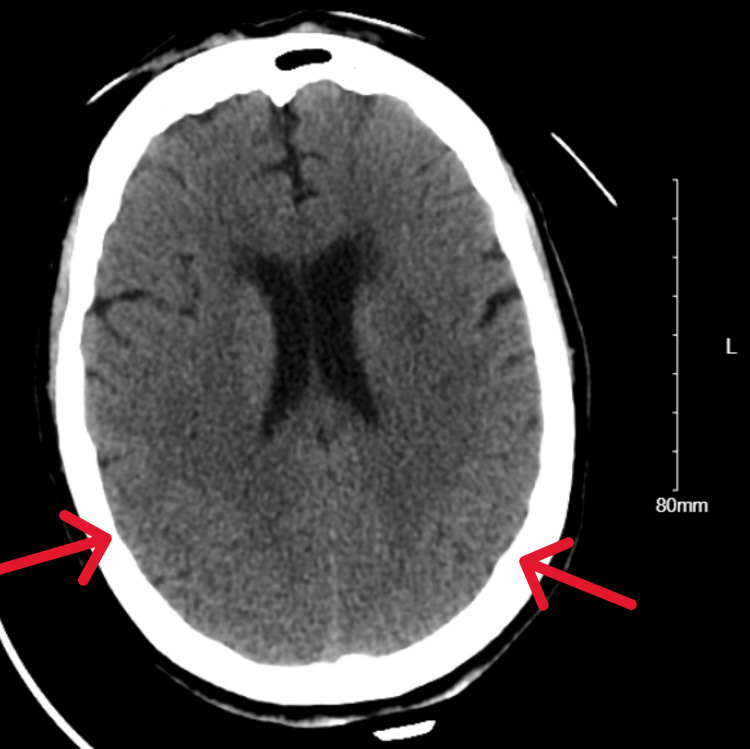
CT of the head w/o contrast showing bilateral sulci effacement CT: computed tomography

A 68-year-old female patient with a past medical history of anxiety, attention-deficit/hyperactivity disorder, and asthma presented to the emergency department with altered mental status and seizure-like activity. She was found by her spouse on the floor alongside a bottle of dicyclomine. The patient displayed confusion, hallucinations (attempting to grasp imaginary objects), dilated pupils, urinary retention, blue-stained tongue (attributed to dicyclomine's color), low-grade fever (37.7°C), tachypnea, and mild tachycardia (heart rate of 101 beats per minute). Upon discussion with the family, it was revealed that the patient was taken off alprazolam approximately one week prior by her primary care physician and was prescribed dicyclomine. According to the patient, dicyclomine was prescribed to help with benzodiazepine withdrawal. The prescription was filled the day prior to hospitalization (30-day supply, 10 mg tablets). We were unable to identify the prescribing physician's rationale for the dicyclomine prescription. The patient was taking dicyclomine frequently because she believed the dicyclomine produced the same effects as a benzodiazepine. It is unknown exactly how much dicyclomine she ingested. Investigations revealed a serum sodium level of 120 mmol/L, a magnesium level of 1.5 mmol/L, a creatine kinase (CK) level elevated to 1,688 U/L, and a normal sinus rhythm on electrocardiogram (EKG) without ischemic changes, widened QRS, or prolonged QTc interval (Figure 1 and Figure 2). Computed tomography (CT) scan of the brain shows crowding of sulci over the cerebral hemispheres, which may be physiologic or related to cerebral edema (Figure 3).

**Figure 4:**
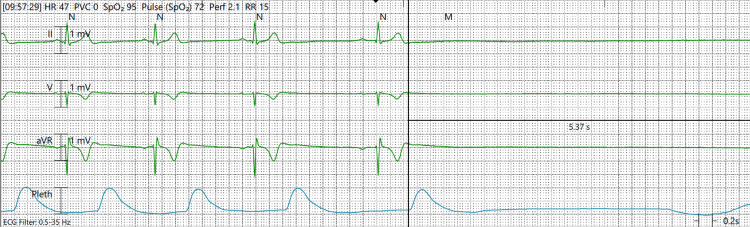
Sinus pause on telemetry

The patient was admitted to the intensive care unit (ICU), received a bolus of intravenous 3% sodium chloride solution, and was started on a half-normal saline infusion to gradually increase the serum sodium level by 6-8 mmol/L over 24 hours (Table 1). Sodium was corrected gradually to prevent the development of osmotic demyelination syndrome. The following day, the patient experienced multiple incidences of prolonged sinus pause, culminating in witnessed asystole lasting 5-10 seconds. Although the sinus pause was unable to be captured on EKG, it was captured on telemetry (Figure 4). The initial episode was around 10:00 am and lasted for five seconds without loss of consciousness. The second episode occurred at approximately 11:30 am and lasted approximately six seconds without loss of consciousness. The third episode was at 11:55 am and lasted for about 10 seconds with the patient jolting her hands in the air and losing consciousness. This event was witnessed by the ICU providers. There was no postictal state noted. By this time, the patient's sodium had improved to 126 mmol/L. Cardiology was consulted and evaluated the patient around 2:00 pm. They noted a similar episode of the patient jolting her hands in the air, loss of consciousness, and asystole on the monitor for 5-10 seconds. A temporary transvenous cardiac pacemaker was then placed by the cardiology team within two hours to prevent serious complications such as heart failure, stroke, and cardiac arrest. The backup rate was set at 50 beats per minute. The patient required pacing multiple times overnight. On hospital day 3, the temporary pacemaker was inadvertently dislodged twice during patient agitation and while utilizing the bedpan, necessitating replacement on each occasion. The patient required lorazepam, haloperidol, and dexmedetomidine infusion for the management of agitation. She continued to require intermittent transvenous pacing on hospital day 4. By this time, her sodium had normalized to 140 mmol/L. Psychiatry was consulted to help determine if the overdose was a suicide attempt. The patient denied any suicidal ideation, and psychiatry determined the patient's overdose was unintentional. On hospital day 5, the temporary pacemaker was removed. No further episodes of sinus pause occurred, facilitating the patient's discharge on the subsequent day. Repeat blood work two weeks after discharge showed a normal white blood cell count, hemoglobin, thyroid-stimulating hormone, free thyroxine, and serum sodium level. As of one year after hospitalization, the patient has not required readmission to the hospital. To our knowledge, the patient has had no further episodes of sinus pause, syncope, or other complications.

**Table 1:** Chemistry profile during hospitalization

	Day 1 am	Day 1 pm	Day 2 am	Day 2 pm	Day 3 am	Day 3 pm	Day 4 am	Day 4 pm	Day 5 am
Sodium (mmol/L)	120	130	126	123	124	133	141	140	140
Potassium (mmol/L)	4.7	3.4	3.4	4.4	4.1	3.7	3.6	3.3	3.3
Chloride (mmol/L)	84	96	92	91	94	100	110	108	107
Creatinine (mg/dL)	0.67	0.68	0.68	0.59	0.61	0.66	0.64	0.66	0.62
Magnesium (mg/dL)	1.5	2.1	2.0	1.9	2.4	-	2.1	-	1.9

## Discussion

An older patient presenting to the emergency department for altered mental status and first-time seizure must be evaluated for a broad differential. In this case, the patient was found with an empty bottle of dicyclomine so there was significant concern for dicyclomine overdose. There were also multiple signs and symptoms present at initial evaluation consistent with anticholinergic overdose such as hallucinations, mydriasis, tachycardia, delirium, and urinary retention. Additionally, the resolution of the arrhythmia with clearance of the dicyclomine was also consistent with anticholinergic toxicity. Other ingestions were considered as well. Blood acetaminophen, salicylate, and ethanol levels were undetectable. Urine drug screen was positive for opiates, benzodiazepines, and cannabis. However, the patient had received midazolam from emergency medical services (EMS) for seizure before this urine sample was obtained. The family reported that the patient no longer had access to her previously prescribed alprazolam. The positive benzodiazepine result was most likely from the appropriate dose of midazolam administered by EMS. Benzodiazepine overdose was therefore unlikely. The patient's symptoms developed a week after the discontinuation of alprazolam. It was therefore believed that the patient was outside of the window for acute benzodiazepine withdrawal. The patient was also prescribed hydrocodone-acetaminophen for chronic pain, which explains the positive screen for opiates. The patient's presentation with dilated pupils and tachypnea was not consistent with the expected miosis and bradypnea expected with opioid overdose. The patient did not have an elevated anion gap metabolic acidosis to suggest ingesting toxins such as methanol, ethylene glycol, isopropanol, valproic acid, topiramate, or lithium. Intracranial pathology was also considered. CT of the brain without contrast revealed crowding of convexity sulci, which may be physiologic or related to cerebral edema. Repeat head CT the next day showed no change in the degree of crowding of the sulci over the cerebral hemispheres. The patient also had no known cause for cerebral edema. Therefore, it was presumed these CT findings were more likely physiologic and less likely due to true cerebral edema. Endocrine and electrolyte abnormalities were also considered. However, blood glucose and thyroid-stimulating hormone levels were within normal limits. The patient was noted to have hyponatremia (serum sodium 120 mmol/L) and mild hypomagnesemia (serum magnesium 1.5 mmol/L) at the initial presentation. Hyponatremia is a common cause of altered mental status and seizure. There are also case reports documenting patients with sinus pause or other cardiac electrical conduction abnormalities in the setting of hyponatremia and hyperkalemia [8-10]. In that case, the patient had a serum sodium level of 102 mmol/L correlating with sinus pauses and junctional escape rhythm during 24-hour Holter monitoring [8]. Hyperkalemia (serum potassium of 6 mmol/L) introduced a potential confounding factor in that case. However, it was felt that electrolyte abnormalities did not contribute to our patient's intermittent sinus pause as the arrhythmia continued after the patient's electrolytes normalized. For example, the patient continued to require intermittent transvenous pacing on hospital day 4. At that time, the serum sodium level was within normal limits at 140 mmol/L and had been within normal limits for over 24 hours. Additionally, most cases of hyponatremia-induced arrhythmia and seizure occur at sodium levels less than 110-115 mmol/L, much lower than our patient's initial sodium. An infection could also explain the patient's mild tachycardia, low-grade fever, and leukocytosis (white blood cell count of 19.3×10^9^/L). However, the patient's procalcitonin level was within normal limits, suggesting a low likelihood of bacterial infection. The patient also had no infectious symptoms to suggest viral or bacterial infection prior to the sudden onset of altered mental status. Finally, cardiac pathology was also considered. The patient's EKG showed no acute ischemic changes, and serum troponin was not elevated. The echocardiogram showed normal left ventricular systolic function with an estimated ejection fraction of 65-70%. There were normal diastolic function, no regional wall motion abnormalities, and no significant valvular abnormalities. Sick sinus syndrome could have explained the patient's arrhythmias but was deemed unlikely as the patient had no preceding history suggestive of sick sinus syndrome such as syncope, dizziness, palpitations, or pre-syncope. Additionally, the episodes of sinus pause resolved by hospital day 5 and have not recurred since. The patient's medication history was unremarkable for agents affecting sinoatrial (SA) node function or heart rate modulation. All medical teams involved in the patient's care concurred that the observed episodes of sinus pause most likely stemmed from anticholinergic toxicity, supported by the self-resolving nature of symptoms and corroborating history.

## Conclusions

Tachycardia typically constitutes a cardiovascular sequela of anticholinergic toxicity. Intriguingly, the intermittent sinus pauses observed in this case garnered consensus among all involved services (critical care, internal medicine, and cardiology) attributing the phenomenon to anticholinergic overdose (dicyclomine). Alternative etiologies were considered but deemed incongruous with the patient's clinical presentation. While the occurrence of sinus pause in the context of anticholinergic overdose remains rare, it underscores the importance of recognizing its potential contribution to life-threatening arrhythmogenic events, warranting heightened clinical awareness and further research. Clinicians should consider anticholinergic toxicity as a potential cause of intermittent sinus pause, especially in patients taking anticholinergic medications. Increased clinical vigilance could impact treatment decisions, including potentially avoiding unnecessary procedures such as permanent pacemaker placement, if symptoms resolve with cessation of the offending agent.
